# Blood Compatibility of Drug–Inorganic Hybrid in Human Blood: Red Blood Cell Hitchhiking and Soft Protein Corona

**DOI:** 10.3390/ma16196523

**Published:** 2023-09-30

**Authors:** Jing Xie, Hyoung-Mi Kim, Kai Kamada, Jae-Min Oh

**Affiliations:** 1 Department of Energy and Materials Engineering, Dongguk University-Seoul, Seoul 04620, Republic of Korea; jingxie@dongguk.edu; 2 Biomedical Manufacturing Technology Center, Daegyeong Division, Korea Institute of Industrial Technology (KITECH), Yeongcheon-si 38822, Republic of Korea; annabb@kitech.re.kr; 3 Department of Materials Science and Engineering, Faculty of Engineering, Nagasaki University, 1-14 Bunkyo-machi, Nagasaki 852-8521, Japan

**Keywords:** blood compatibility, biocompatibility, layered double hydroxide, drug-delivery system, methotrexate, red blood cell, human plasma, hitchhiking, protein corona

## Abstract

A drug-delivery system consisting of an inorganic host—layered double hydroxide (LDH)—and an anticancer drug—methotrexate (MTX)—was prepared via the intercalation route (MTX-LDH), and its hematocompatibility was investigated. Hemolysis, a red blood cell counting assay, and optical microscopy revealed that the MTX-LDH had no harmful toxic effect on blood cells. Both scanning electron microscopy and atomic force microscopy exhibited that the MTX-LDH particles softly landed on the concave part inred blood cells without serious morphological changes of the cells. The time-dependent change in the surface charge and hydrodynamic radius of MTX-LDH in the plasma condition demonstrated that the proteins can be gently adsorbed on the MTX-LDH particles, possibly through protein corona, giving rise to good colloidal stability. The fluorescence quenching assay was carried out to monitor the interaction between MTX-LDH and plasma protein, and the result showed that the MTX-LDH had less dynamic interaction with protein compared with MTX alone, due to the capsule moiety of the LDH host. It was verified by a quartz crystal microbalance assay that the surface interaction between MTX-LDH and protein was reversible and reproducible, and the type of protein corona was a soft one, having flexibility toward the biological environment.

## 1. Introduction

Layered double hydroxides (LDHs) have recently attracted particular attention as efficient drug-delivery systems (DDSs). They are known to stabilize biologically unstable drug molecules to protect them [1], enhance the solubility of less soluble drugs [2,3], release the incorporated moiety in a controlled manner [4], effectively internalize into the target cell [5,6], and to have low cytotoxicity [7]. Based on the extensive studies on LDH-based DDS both in vitro and in vivo [8,9,10], recently, it was suggested that delivery of anticancer agents to a target organ was possible in animal models [11,12,13]. Although various injection routes, such as oral [14], intraperitoneal [15], intertumoral [16], intravenous administration [17], etc., have been utilized, most of the studies adopt intravenous administration as a practical and effective method. In order to optimize the intravenous administration of a certain drug-delivery system, its blood compatibility should be elucidated. Despite the growing interest in LDH as a DDS, there has been only limited information on the hematocompatibility of LDH. We recently reported that LDH itself demonstrated negligible blood cell damage regardless of the particle size surface charge [18] and that LDH did not seriously denature the plasma proteins [11,19]. Other research also reported that surface modification of LDH with polysulfobetaine had excellent blood compatibility [20,21]. However, to the best of our knowledge, there has been only limited information on the hemocompatibility for drug-incorporated LDH, and it is not clear where the compatibility originates from.

Recent studies have suggested that the nanoparticles interact briskly with protein moieties when they encounter biological systems [22,23,24]. The protein adsorption on nanoparticles mutually influences each other, determining both the colloidal properties of drug-delivery nanoparticles and the fate of biological moieties [25,26]. Protein corona, which is referred to as a thin protein layer formed on nanoparticles through moderate interaction, is considered a strategy of nanoparticle DDS to acquire biocompatibility and high delivery efficiency. Hu et al. revealed that the protein corona formed in the surrounding of graphene oxide nanoparticles could reduce the cytotoxicity [27]. The formation of protein corona around gold nanoparticles [28] and carbon nanotubes [29] was reported to facilitate loading and release of drugs. Landgraf et al. presented that the gold nanoparticle-incorporated Fe_3_O_4_ with protein corona had enhanced biocompatibility in mouse systems compared with corona-free ones [30]. Meanwhile, we previously reported that LDH nanomaterials were fairly inert to red blood cells (RBCs) regardless of the size and surface charge [18], and that LDH could interact with proteins to different degrees depending on the size and surface charge [19]. The drug-incorporated LDH, of which the surface might be partially covered by the drug, would undergo different blood interactions [11]. 

In this study, an anticancer drug, methotrexate (MTX), incorporating LDH (MTX-LDH), was selected to examine the blood compatibility of an LDH-based DDS. MTX is an antineoplastic agent and has been widely utilized to treat various solid and liquid tumors, including breast cancer, head and neck cancer, leukemias, lung cancer, lymphomas, osteosarcoma, etc. The cellular internalized MTX binds to dihydrofolate reductase and inhibits de novo purine synthesis, giving rise to the death of cancer cells [31,32]. Despite its clear action mechanism, its low cellular uptake and lack of selectivity render the need for high-dose administration of MTX, and thus, various side effects, such as nausea, fever, low white blood cell counts, and breakdown of the skin, have been seen [33]. In this regard, the effective delivery of MTX to lesions has been one of the major topics in drug-delivery research, and the MTX-LDH hybrid was selected as one of the solutions both in vitro and in vivo [34,35,36,37].

**Scheme 1:**
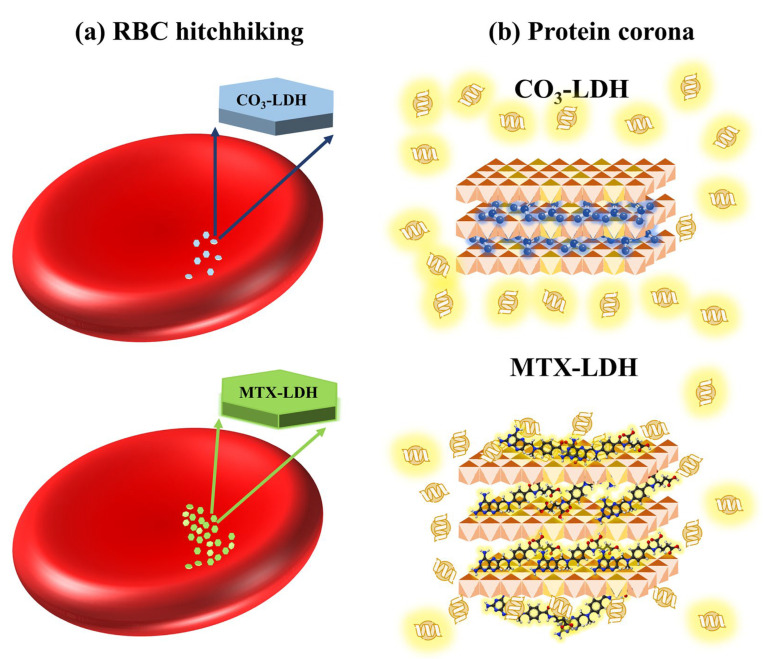
Schematic illustration of LDH interacting with RBCs (**a**) and plasma protein (**b**).

Taking into account the previous studies on LDH alone, the blood compatibility of MTX-LDH was researched in terms of the blood cell interaction and protein interaction. As we reported previously [18], we hypothesized that the MTX-LDH hybrid can also take advantage of RBC hitchhiking and the protein corona effect (Scheme 1). The RBCs’ interaction with MTX-LDH was investigated by hemolysis and the cell counting assay. The morphological change in blood cells and the potential interaction between MTX-LDH and cells were visualized by optical microscopy, electron microscopy, and atomic force microscopy (AFM). In addition, the interaction between plasma protein and MTX-LDH was investigated by evaluating the colloidal properties of MTX-LDH in the plasma condition. The quantitative interaction between MTX-LDH and proteins was analyzed by the fluorescence quenching assay, and the quartz crystal microbalance techniques were applied to comprehend the interaction. 

## 2. Materials and Methods

### 2.1. Materials

Magnesium nitrate hexahydrate (Mg(NO_3_)_2_·6H_2_O, 99%), aluminum nitrate nonahydrate (Al(NO_3_)_3_·9H_2_O, ≥98%), sodium bicarbonate (NaHCO_3_, 99%), the anticancer drug methotrexate (MTX), and albumin solution human (A9080) were purchased from Sigma–Aldrich Co., LLC. (St. Louis, MO, USA). Sodium hydroxide pellets (NaOH, ≥98%) were obtained from Daejung Chemicals and Metals Co., Ltd. (Siheung, Republic of Korea). In this study, whole blood was obtained from a healthy volunteer under the approval of Yonsei University Wonju College of Medicine (Approval No. YWMR-12-6-030). All the chemicals were used without further purification.

### 2.2. Synthesis of MTX-LDH and LDH without Drug Moiety

To synthesize the anticancer drug-incorporated LDH hybrid (MTX-LDH) using the co-precipitation route [38,39], powdered MTX was first dissolved in decarbonated water and titrated with NaOH (0.5 M) solution to yield a clear yellowish solution at pH 7. A mixed-metal solution containing Mg(NO_3_)_2_·6H_2_O (0.032 M) and Al(NO_3_)_3_·9H_2_O (0.016 M) (2:1 molar ratio, in decarbonated water) was added to the MTX solution. The reactant was then titrated with 0.9 M NaOH (decarbonated water) until pH ~ 9.5 to produce the yellow suspension. The suspension was stirred under N_2_ atmosphere for 48 h and then separated by centrifugation (6000 rpm, 5 min). Unreacted salts were thoroughly washed with decarbonated water, and the sample was stored in the filter cake state.

In order to prepare LDH without the MTX moiety, a conventional co-precipitation method was adopted [40,41]. A mixed-metal solution containing Mg(NO_3_)_2_·6H_2_O (0.032 M) and Al(NO_3_)_3_·9H_2_O (0.016 M) (2:1 molar ratio) was titrated with 0.9 M NaOH containing 0.647 M NaHCO_3_ until pH ~9.5. The white suspension was stirred under ambient conditions for 48 h. After the reaction, the precipitate was separated by centrifugation (6000 rpm, 5 min), thoroughly washed with deionized water, and stored in the filter cake state.

### 2.3. In Vitro Biological Assay: Hemolysis Assay and Red Blood Cell Counting

In order to evaluate the interaction between MTX-LDH and blood components, the harmful effect on red blood cells (RBCs) was assessed by hemolysis and the cell counting assay with or without contact with the MTX-LDH suspension. Whole blood was used for both assays. The hemolysis assay was carried out following the previous reports [11,18,42] by modifying the American Society for Testing and Materials [43]. The MTX-LDH filter cake was suspended in saline (0.9% NaCl solution) to yield concentrations of 1, 5, and 10 mg/mL. In order to prepare a homogeneous suspension, the designated amount of MTX-LDH powder was added to saline, and the mixture was magnetically stirred for 10 min and sonicated with a bath ultrasonicator for 1 min. If the suspension contained large and heterogeneous particles perceptible with the naked eye, the mixing process of stirring and sonication was repeated. After the suspension was homogeneously prepared, it was directly subjected to in vitro assays. The deionized water and saline were used as positive (100% hemolysis) and negative controls (0% hemolysis), respectively. As the MTX content in MTX-LDH was ~44.5 wt%, the dose of MTX was approximately 0.45, 2.25, and 4.5 mg/mL, respectively. Blood and MTX-LDH suspension were mixed in a 1:1 volume ratio, then gently incubated at 36.5 °C. After 0.5 and 24 h, the mixture was placed at room temperature for another 0.5 h to change hemoglobin to oxyhemoglobin. The samples were gently centrifuged at 3000 rpm for 5 min to separate blood cells, and the absorbance of the supernatant at 540 nm was measured by a UV-vis spectroscope (SHIMADZU UV-1800, Shimadzu Schweiz GmbH, Reinach BL, Switzerland). The blood with deionized water instead of saline and the one collected at 0 h were utilized as positive (100% hemolysis) and negative controls (0% hemolysis), respectively.

The number of viable blood cells after MTX-LDH contact was checked with a hemacytometer (Sigma–Aldrich, St. Louis, MO, USA) [44]. Whole blood was treated with MTX-LDH suspension, as in the hemolysis assay, and was diluted 200 times with a 0.85% sodium chloride solution. The number of cells in a chamber (9 mm^2^) of the hemacytometer was determined by 5 times repeated counting.

### 2.4. Microscopy Studies

The morphology of blood cells and the possible interaction between MTX-LDH and blood cells were visualized with optical microscopy (OM, Korea lab tech, Seong-Nam, Republic of Korea) and scanning electron microscopy (SEM, FEI QUANTA FEG250, Hillsboro, WA, USA). The blood was treated with MTX-LDH suspension, as in the hemolysis assay. A drop of the mixture was located on the slide glass, smeared, fixed with methyl Wright’s stain solution (pure chemicals, Tokyo, Japan) for 4 min, and stained with Wright’s stain buffered at pH 6.4 for 6 min. The slides were directly subjected to optical microscopy in order to check the morphological change in blood cells. For the SEM study, the slides were sputtered with Pt for 20 s prior to the measurement.

The surface interaction between MTX-LDH particles and RBCs was cross-confirmed with atomic force microscopy (AFM, Park Systems NX10, Park Systems Corp., Suwon, Republic of Korea). The blood sample was placed on a glass slide for immobilization, and a 1 × 1 cm piece was cut out. The cut piece was then directly mounted onto the AFM holder. The measurements were conducted in non-contact mode with a scanning rate of 0.5 Hz. The topographic images were processed and analyzed using XEI 4.3 software (Park Systems Corp, Suwon, Republic of Korea), and a smoothing filter was applied to remove the low-frequency noise in the scanning directions.

### 2.5. Colloidal Properties of MTX-LDH in Human Plasma

In order to evaluate the interaction between MTX-LDH and human plasma, the colloidal behaviors of MTX-LDH were investigated by dynamic scattering (DLS) and electrophoretic light scattering (ELS) measurements to determine the hydrodynamic diameter (*d*) and zeta potential (*ζ*), using the Otsuka Electronics ELSZ-1000 (Otsuka Electronics, Osaka, Japan) [6]. The MTX-LDH was suspended in either a neutral aqueous condition or human plasma to reach a 0.1 mg/mL MTX-LDH concentration. After 0.5 h and 6 h of incubation, the colloidal properties of MTX-LDH in each condition were monitored by measuring the hydrodynamic radius and zeta potential.

### 2.6. Human Plasma Fluorescence Quenching

In order to assess the potential interaction between MTX-LDH and protein, plasma was obtained from the whole blood via centrifugation at 3000 rpm for 5 min. The supernatant was diluted with Ca^2+^/Mg^2+^-free Dulbecco’s phosphate-buffered saline (DPBS) 70 times. Two kinds of suspensions—MTX-LDH and LDH—and the MTX solution were prepared in DPBS. The samples were mixed with the plasma solution at a 1:1 volume ratio to obtain sample concentrations of 0, 0.2, 0.4, 0.6, 0.8, 1.0, 1.5, and 2.0 mg/mL. The mixtures were sonicated for 1 min and placed on a thermo-finemixer (FINEPCR SH2000-DX, FINEPCR, Gunpo, Republic of Korea) at 36.5 °C for 30 min. Then, the mixtures were centrifuged at 15,000 rpm for 5 min, and the fluorescence intensity, at 340 nm, of the supernatants, with an excitation wavelength of 280 nm, was measured using a luminescence spectrometer (Perkin Elmer LS55, PerkinElmer, MA, USA) [45,46]. Quenching ratios were calculated as the ratio: (*F*_0_ − *F*)/*F*_0_, where *F*_0_ and *F* represent the fluorescence intensity of the negative control and the quencher-treated samples, respectively. 

In order to determine the fluorescence quenching nature (i.e., static and/or dynamic interactions), different mathematical models were applied for the MTX-, MTX-LDH-, and LDH-treated human plasma.

A completely dynamic fluorescence quenching mechanism was described as the classical Stern–Volmer equation [47,48,49]:

(1) $$\frac{F_{0}}{F}=1+K_{SV}\left[C\right]$$

where [*C*] is the quencher concentration, *K_SV_* is the Stern–Volmer constant, and *F* and *F*_0_ are, respectively, the fluorescence intensity in the presence and absence of the quencher. In this case, a linear plot of *F*_0_/*F* vs. the quencher concentration should occur with an intercept value of 1.0.

For a wholly static quenching mechanism [50], the experimental data generally fit the Perrin equation:

(2) $$Log\frac{F_{0}}{F}=K_{S}\left[C\right]$$

where *K_S_* is the static quenching constant. Different from the Stern–Volmer equation, the *F*_0_/*F* ratio is not consistently linearly with the quencher concentration and presents an upward curvature at a high quencher concentration.

For the combined dynamic and static quenching, the following polynomial equation can be applied [51,52]:

(3) $$\frac{F_{0}}{F}=1+\left(K_{SV}\left[C\right]+K_{SV}K_{S}{\left[C\right]}^{2}\right)$$

where *K_SV_* and *K_S_* are the dynamic (Stern–Volmer) and static quenching constants, respectively. When *F*_0_/*F* is plotted against [*C*], an upward curvature is observed. The *K_SV_* + *K_S_* values can be obtained from curve fitting and Equation (3). 

The graphs representing the Stern–Volmer and Perrin equations, as well as the above-mentioned polynomial equation, were obtained using Microcal Origin 6.0 professional software.

### 2.7. Evaluation of Interaction between MTX-LDH and Protein by Quartz Crystal Microbalance (QCM)

The surface interaction between MTX-LDH particles and human blood plasma protein was quantitatively analyzed via the QCM technique using a frequency analyzer (QCA917-20, Seiko EG&G Co., Tokyo, Japan). An AT-cut quartz plate with Au coatings on both sides (QA-A9M-Au, Seiko EG&G Co., Tokyo, Japan), with a fundamental resonance frequency of 9 MHz, was utilized as a QCM tip. The surface of the Au film was coated with MTX-LDH by casting, and the vibration of the tip was initially equilibrated in stirred pure water. Subsequently, alternating injections of a 10 mg/mL human serum albumin solution and pure water were performed, and the frequency change was monitored.

## 3. Results and Discussion

### 3.1. In Vitro Blood Compatibility of MTX-LDH

**Figure 1:**
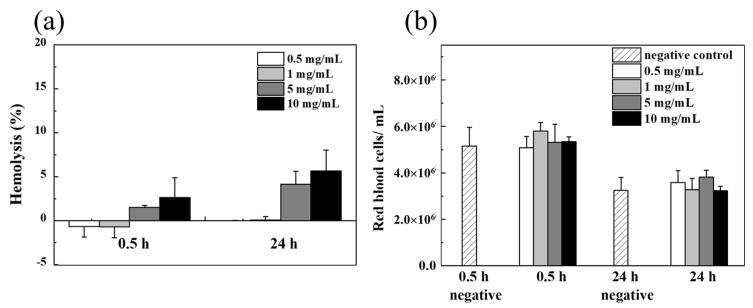
In vitro (**a**) hemolysis and (**b**) the number of viable blood cells after incubation with MTX-LDH suspension with 0.5 mg/L, 1 mg/mL, 5 mg/mL, and 10 mg/mL for 0.5 h and 24 h in human blood.

The biocompatibility of MTX-LDH towards human blood was evaluated by both the hemolysis and cell counting assays, as shown in Figure 1. Notably, the hemolysis percentages of MTX-LDH-treated human blood were less than 1% after incubation for 24 h at concentrations of 0.5 mg of MTX-LDH/mL and 1 mg of MTX-LDH/mL. The hemolysis ratio was significantly lower than the prescribed drug standard and the permissible level for blood-contacting biomaterials, 5% [53], suggesting the high biocompatibility of MTX-LDH in the blood system. In addition, Rahman et al. found that the hemolysis of human RBCs treated with 0.45 mg/mL of MTX for only 4 h was 35% [54]. The reduced hemolysis of MTX-LDH strongly suggested that the blood interaction of MTX was greatly suppressed by the encapsulation with LDH, as LDH nanoparticles exhibited negligible hemolytic activity of RBCs treated with LDH, with concentrations of 1 mg/mL, 5 mg/mL, and 10 mg/mL, which was less than 1%, as shown in Figure S1. Although MTX-LDH at a very high concentration of 10 mg/mL gave rise to slightly higher hemolysis of 6% standard after incubation for 24 h, compared with the permissible limits, this was not interpreted as a harmful effect of MTX-LDH. In the clinical treatment of several cancers, the desired dosage of MTX for adults is ~0.8 mg/kg [55]. Taking into account that an adult has a body weight of ~70 kg, and ~5 L of blood, the clinically encountered concentration of MTX in the body is 0.05 mg/mL, which is converted to a concentration of MTX-LDH 0.12 mg/mL. The MTX-LDH showed only slight hemolysis at a 40 times higher concentration than the clinical condition; thus, we could suggest that the toxic effect of MTX-LDH on human red blood cells was negligible. Furthermore, we could not observe any statistically significant hemolysis at all the tested concentrations at 0.5 h.

To further cross-confirm the blood compatibility of MTX-LDH, the number of viable blood cells after treatment with MTX-LDH was counted using a hemacytometer (Figure 1b). There was no distinctive difference in viable blood cells regardless of the incubation time and MTX-LDH concentration. In the previous report, Woolley et al. reported that the percentage of survival of RBCs was 0% at 4 h after treatment with 4.5 mg of MTX/mL in vivo using a ^51^Cr-labeled RBC method [56]. This revealed that MTX drugs have cytotoxicity toward RBCs. However, the number of blood cells after treatment with LDH and MTX-LDH nanoparticles with the doses of 1 and 10 mg/mL almost did not decrease compared to the negative control (Figure S1), suggesting that the cytotoxicity of MTX could be inhibited by covering the nanoparticles. Even at the 10 mg/mL MTX-LDH dosage, at which hemolysis of 6% was detected, the number of viable cells did not statistically decrease. These results strongly suggested that MTX-LDH did not have a lethal effect on blood cells, even though MTX-LDH induced slight damage on the cell membrane at a very high concentration. 

### 3.2. Investigation of the RBC Morphology

**Figure 2:**
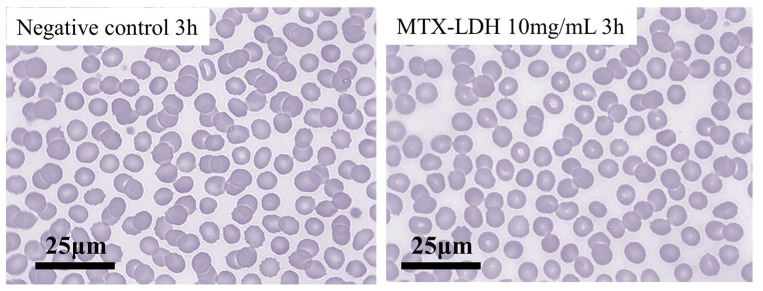
Optical microscopic image of RBCs in human blood untreated/treated with MTX-LDH (10 mg/mL) after 3 h of incubation.

In order to cross-confirm the blood toxicity of MTX-LDH, the change in the apparent shape of RBCs upon contact with MTX-LDH was monitored by an optical microscope (Figure 2). No significant abnormal shape of RBCs was observed after 3 h of incubation with a very high concentration of MTX-LDH (10 mg/mL). A comparison between the MTX-LDH-treated blood and the negative control group clearly showed that the disc-type shape of RBCs did not change, revealing that MTX-LDH did not physically harm the RBCs. This meant that the MTX-LDH did not have incidence in terms of blood pathologies even at a high concentration of 10 mg/mL, since abnormal morphologies of RBCs related to the pathological findings were not observed [57]. It has been frequently reported that the several materials that are under consideration as drug-delivery carriers result in size and shape changes of RBCs. Avsievich et al. found that the smooth outline of RBCs changed to a sea urchin-like morphology—echinocyte—when RBCs were incubated with 0.1 mg/mL of nanodiamonds colloid in phosphate-buffered saline (PBS) due to the strong interaction through the surface charge effect [58]. Chwalibog’s group studied the interaction between RBCs, and several graphitic materials and they discovered morphological changes and significant lysis of RBCs after contact with graphene, graphene oxide, and reduced graphene oxide [59]. Specifically, the RBCs treated with graphitic materials with concentrations of 0.05–5 mg/mL in PBS suffered from morphological changes to a spiked surface (echinocyte) or double-concave form (knizocytes), suggesting the acute toxicity of those materials [59]. As the MTX-LDH did not induce any significant morphological change in RBCs even with a very high concentration (10 mg/mL), we could temporarily suggest that MTX-LDH is advantageous compared with other drug-delivery materials in terms of blood compatibility. This property supports MTX-LDH as an intravenously injectable drug-delivery system.

### 3.3. Nanoparticle Attachment on the Surface of RBCs

**Figure 3:**
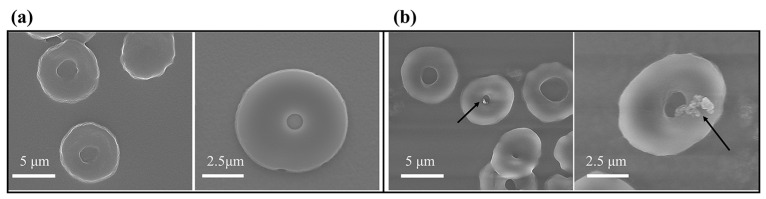
The SEM images of (**a**) RBCs alone (negative control) and (**b**) RBCs treated with MTX-LDH (10 mg/mL) for 3 h. The MTX-LDH particles on the surface of RBC are indicated by the black arrows.

From the in vitro assays, it was confirmed that the MTX-LDH had high biocompatibility toward RBCs. At this stage, we could suggest two explanations: (1) the MTX-LDH particles do not interact physically with RBCs and thus had no chance to harm the cells directly, and (2) the MTX-LDH particles can contact RBCs, but the degree of interaction is moderate and minimized physical attacks to the cells. In order to address the above questions, the localization of MTX-LDH after treatment with human whole blood was investigated by SEM measurement (Figure 3). It was clearly shown that some particles, which are considered MTX-LDH according to the particle size and shape (Figure S2), were attached to the surface of RBCs (Figure 3b). The morphologies between LDH without the MTX moiety and MTX-LDH were almost identical (Figure 3b), as the mean particle sizes of LDH without MTX and MTX-LDH were 167 ± 37 and 156 ± 49 nm, respectively. The LDH and MTX-LDH both presented a typical plate-like shape (Figure S2). In our previous study [18], LDH nanoparticles did not tend to attach to the surface of RBCs. However, MTX-LDH nanoparticles were adsorbed on the surface of RBCs, as shown in Figure S3, in which ~90% of RBCs carried MTX-LDH nanoparticles without disrupting the RBCs. Therefore, it was suggested that most of the RBCs were involved in the hitchhiking process of the MTX-LDH drug-delivery system [60,61]. A similar pattern of particle attachment on RBCs was previously reported as RBC hitchhiking [60,62,63]. Generally, this kind of non-covalent attachment of a nanocarrier in this manner is chosen as a strategy to boost the delivery effect of nanocarriers to target organs by dramatically enhancing the systemic circulation [64]. It should be noted here that the attachment of a nanocarrier on RBCs is not always advantageous in drug delivery. For example, 0.1 mg/mL of SBA-15-type mesoporous silica nanoparticles in PBS were reported to adsorb effectively on the surface of RBCs; however, the strong interaction gave rise to the disturbance of the membrane and morphological changes of RBCs [65]. As the MTX-LDH showed negligible hemolysis, no significant morphological change in RBCs, and even an RBC hitchhiking effect, it could be suggested as a highly biocompatible and long-circulating drug-delivery system. 

**Figure 4:**
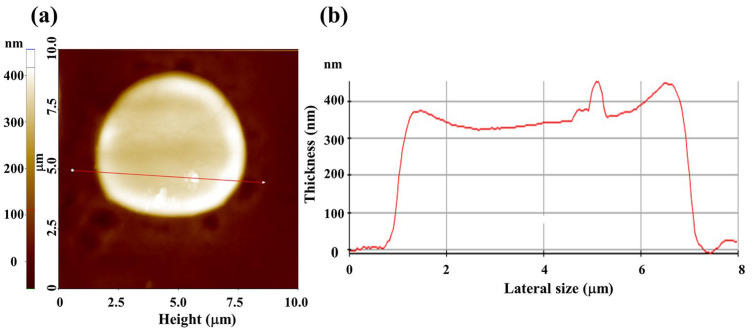
(**a**) AFM images of red blood cells in human blood as a morphology map, and (**b**) cross-sectional height profile of the line in the image in (**a**).

In order to cross-confirm the RBC hitchhiking effect of MTX-LDH, the surface topology of MTX-LDH-attached RBCs was visualized with AFM. As shown in Figure S4, the MTX-LDH particles had a ~150 nm diameter and ~20 nm thickness. From the multiple steps in the topology, we could expect that these particles were an agglomerate of smaller particles. Since the particle size of ~150 nm was reported as the optimum condition for LDH in terms of cellular uptake and retention [5], the current MTX-LDH was considered appropriate for anticancer drug-delivery systems. The AFM image and surface topology in Figure 4 showed two representative features: (i) the concave disc shape of RBCs and (ii) small particles attached to the surface of the RBCs. The dimension—lateral size of 6~8 μm and a thickness of ~400 nm—as well as the concave disc shape and smooth surface, are evidence of normal RBCs. It means that the MTX-LDH treatment did not induce serious toxic effects on the blood cells. We observed an object of approximately 500 nm in diameter and 100 nm thickness on the concave part of the RBCs. Considering the dimensions of MTX-LDH (Figure S4), the particles may be attributed to agglomeration of MTX-LDH. It is expected that the MTX-LDH adsorbed proteins and made moderate-sized agglomerates. Different from the MTX-LDH particles alone, the protein that was adsorbed on the particles softly mediated the particle–RBC interaction to preserve the nature of the cells. The interaction between plasma protein and MTX-LDH will be discussed in the next section.

### 3.4. Colloidal Stability of MTX-LDH in Human Plasma

Since MTX-LDH is considered an intravenously injectable drug-delivery system, it is important to monitor its colloidal properties, such as the surface charge and hydrodynamic radius. All the intravenously administered colloidal particles interact with plasma protein through a process called opsonization, to determine their in vivo fate [66]. Depending on the colloidal behavior, some particles are readily removed during blood circulation, some exist in the blood vessels for a long time for efficient delivery, and some are strongly aggregated to block the blood vessels. In other words, only an appropriate interaction between colloidal particles and plasma components guarantees the success of the intravenous drug-delivery system. 

**Figure 5:**
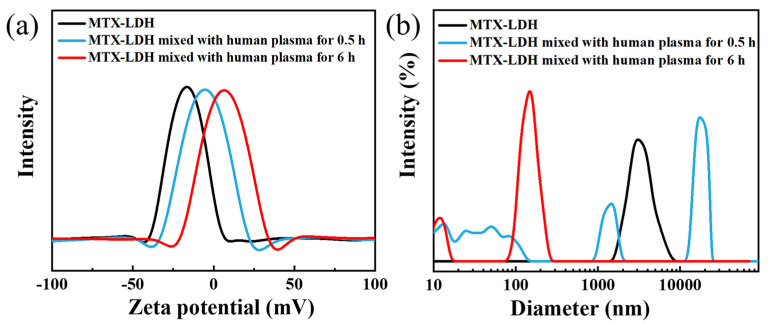
(**a**) Zeta potentials and (**b**) hydrodynamic size distribution of MTX-LDH in neutral aqueous suspension (black) and mixed with human plasma after 0.5 h (blue) and 6 h (red). The concentration of the sample suspension was set as 0.1 mg/mL.

The zeta potential of MTX-LDH in a neutral aqueous condition was −17.0 ± 0.4 mV (Figure 5a), while LDH without the MTX moiety had a positive value of 31.4 ± 0.6 mV (Figure S5). The surface charge of LDH is known to be positive due to the substitution of Al^3+^ for Mg^2+^ in the layer [67,68,69]. The negative zeta potential of MTX-LDH was accountable for the surface adsorption of negatively charged MTX moieties. After the incubation with human plasma, the zeta potential value gradually shifted to the positive direction, finally reaching 7.6 ± 0.9 mV at 6 h. Taking into account the isoelectric points of plasma proteins (4.7, 5.8, and 7.2 for albumin, fibrinogen, and globulin, respectively), the gradual shift of the surface charge from negative to positive was attributed to the surface adsorption of partially positive proteins (e.g., globulin) at the surface of MTX-LDH. 

This hypothesis was also confirmed by the hydrodynamic size change in MTX-LDH. As shown in Figure 5b, the hydrodynamic sizes of MTX-LDH were fairly large (~3000 nm) in aqueous suspension with high heterogeneity, with a polydispersity index of 0.9. This revealed that the MTX-LDH particles are agglomerated in a wide size range. The size distribution curve for MTX-LDH was almost identical to the profile of LDH (Figure S5), suggesting that the colloidal behavior of LDH was not affected by the intercalated MTX moiety. After 0.5 h of incubation with plasma, both large-sized peaks (at ~20,000 nm) and small-sized peaks (below 200 nm) appeared in an uncontrolled manner. This suggested that some MTX-LDH particles took advantage of the protein corona and particle separation, while other MTX-LDH particles were more agglomerated due to the sudden increase in ionic strength in the plasma condition. After 6 h, the interaction between MTX-LDH and plasma protein might be stabilized, reaching a reduced average hydrodynamic size (~150 nm) and high homogeneity (polydispersity index of 0.17). It is notable that the hydrodynamic size and the primary particle size (see SEM in Figure S2) were almost identical. The decrease in particle size was considered to be the formation of protein corona surrounding MTX-LDH, which suppressed the inter-particle attraction among MTX-LDH nanoparticles [70,71]. It was thought that the moderate opsonization to form protein corona [72] hindered the non-specific agglomeration to evenly disperse the MTX-LDH particles. Similar colloidal stabilization in nanoparticles by protein corona was reported in Au-doped Fe_3_O_4_, and serious aggregation of the particles was avoided by simply incubating with plasma proteins due to the moderate interaction of the protein corona effect [30].

### 3.5. Fluorescence Quenching

**Figure 6:**
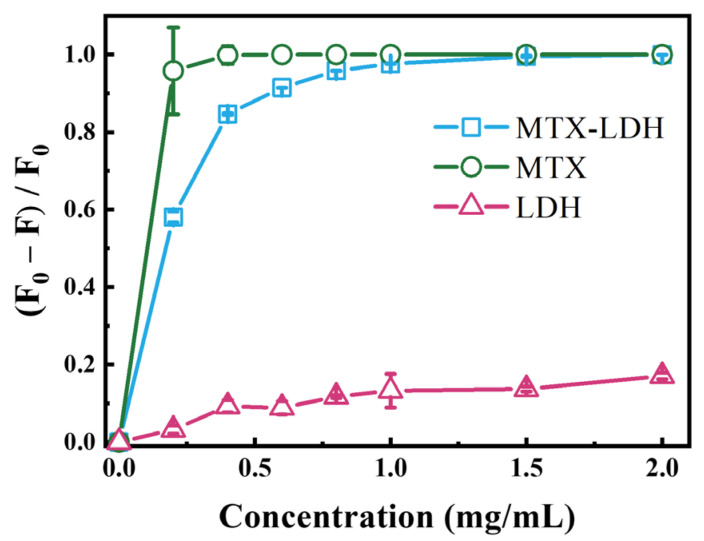
Human plasma fluorescence quenching assay of MTX-LDH, MTX, and LDH, respectively.

In order to quantitatively examine the interaction between MTX-LDH and plasma proteins, the fluorescence quenching assay [73,74,75] was carried out. As the absorption of proteins to the surface of nanoparticles involves a new “biological identity” in the human blood plasma, it determines the successive cellular/tissue reactions [76,77]. As shown in the concentration-dependent fluorescence quenching (Figure 6), MTX-LDH showed saturation quenching at a concentration of 1.5 mg/mL, while LDH nanoparticles had a maximum quenching ratio of less than 0.2, even at very high concentrations. The results represented that the MTX-LDH facilitatedinteraction with human blood plasma compared with pristine LDH nanoparticles. The full fluorescence quenching of MTX was observed at very low concentrations (less than 0.5 mg/mL), demonstrating the strong binding of MTX with plasma protein, as reported previously [78]. Taking into account the strong interaction of MTX and almost inert behavior of LDH, the fluorescence quenching of MTX-LDH could be interpreted as the action of surface-adsorbed MTX on LDH particles [79,80].

To determine the type of quenching occurring in MTX-LDH, the experimental data were fitted to several models, including the Stern–Volmer, Perrin, and polynomial equations [81], as shown in Figure S6 and Table 1. Although we could not fit the quenching pattern of MTX due to the fast equilibrium at very low concentrations, it was obvious that the binding of MTX and protein was categorized as a static type, as recorded from the previous reports [82]. The MTX-LDH was well fitted to both Perrin and polynomial models; on the other hand, LDH was better fitted to the polynomial or Stern–Volmer models. The Stern–Volmer model hypothesizes dynamic quenching, which is caused by a collisional encounter between the fluorophore in protein and the quenchers, whereas the Perrin model is related to the static quenching mediated by the formation of a ground-state complex between the protein and quenchers [83,84]. The polynomial model explains the combination of both dynamic and static. As static quenching assumes a stronger interaction between the protein and quenchers than dynamic quenching, the degree of interaction can be arranged as follows: MTX > MTX-LDH >> LDH. The statistical results of the Perrin and polynomial equations for MTX-LDH proposed dominant control of static quenching, attributed to the formation of ground-state complexes between MTX at the surface of LDH and plasma protein. The dominant Stern–Volmer and polynomial behavior in LDH indicated the major role of dynamic quenching in LDH. It is known that dynamic quenching (Stern–Volmer fitting) is strongly related to the formation of the protein corona. According to the quenching assay, we confirmed that the LDH takes advantage of the protein corona. The MTX-LDH can interact with proteins in both a static (through LDH) and dynamic manner (through surface-adsorbed MTX), giving rise to the protein corona effect. The moderate interaction between MTX-LDH and plasma proteins would be a favorable factor in long-term circulation and facilitate target delivery as a drug carrier. 

In addition to the long-term circulation under systemic conditions, the interaction between MTX-LDH and plasma protein takes the role of drug release suppression. According to the previous research, the MTX release from the MTX-LDH hybrid was influenced by the circumstances. For example, the MTX release was fairly suppressed under the absence of electrolytes, while the increasing ionic strength facilitated drug release [85,86,87]. The intracellular MTX release varies depending on the cellular organ: the lysosomal condition (pH ~4.5 with various enzymes) accelerated drug release compared to the cytosolic condition (pH ~7.4 with electrolytes). In addition to the electrolytes and pH conditions, the existence of protein corona also affects the drug release. As reported previously, the drug release from the superparamagnetic iron oxide nanocarrier was strongly suppressed by the formation of protein corona on it [88]. Obst et al. reported that the presence of protein corona around solid cationic Eudragit RS (EGRS), with ethyl cellulose (EC) nanoparticles loaded with the drugs (dexamethasone), reduced the cytotoxicity and decreased the rate of drug release compared to protein-free EGRS-EC nanoparticles [89]. It is considered that the blocking of exit by the protein moiety retarded the drug release, giving rise to the preservation of loaded drugs under protein corona. Likewise, the moderate interaction between MTX-LDH and plasma protein is thought to suppress the MTX release in blood conditions. Therefore, the formation of protein corona would preserve the MTX moiety in MTX-LDH during blood circulation and enable the specific delivery of MTX when the particle reaches the target organ.

### 3.6. Reversible Adsorption Behavior of Proteins on the MTX-LDH Surface

**Figure 7:**
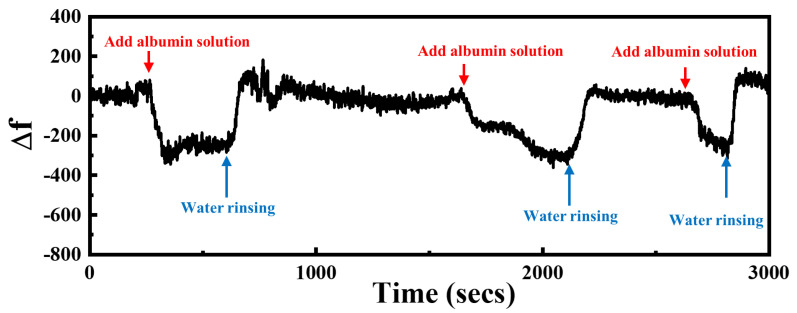
Binding tests using the quartz crystal microbalance (QCM) assay between MTX-LDH and albumin solution. The changes in the frequency (Δf) of the sensor crystal were affected by binding with and without albumin, in which the presence of albumin was adjusted by rinsing with water.

To explore the interaction between plasma proteins and MTX-LDH in detail, the protein adsorption and desorption on MTX-LDH were repeatedly examined using a quartz crystal microbalance (QCM). As shown in Figure 7, the resonance frequency shift (Δf) of MTX-LDH-coated quartz dropped upon the flow of albumin solution, showing that a certain amount of protein was adsorbed on the MTX-LDH. A reduction in the resonance frequency, reaching a maximum shift of approximately 300 Hz, was observed, which was attributed to the saturation of protein adsorption on the MTX-LDH nanoparticles. The frequency shift was maintained, and readily recovered the original value by rinsing with water. The frequency drop and recovery were reversible and reproducible, demonstrating the reversibility of protein adsorption on the MTX-LDH nanoparticles. This kind of fast and reversible adsorption–desorption of proteins on nanoparticles could be interpreted as soft corona [90,91]. The protein corona in nanomedicine can be classified into hard and soft. The former is not easily detached and has a longer lifetime; on the other hand, the soft corona has fast kinetics, and it is more flexible to the new environment. The QCM results implied that the MTX-LDH particles might have soft protein corona in the intravenous condition, so that they can flexibly cope with the biological events without causing harm to blood components. In fact, this has been demonstrated with various polymer materials, such as dextran-doped-polypyrrole [92] and a polyethyleneoxide/polyacrylicacid mixture [93], where they were not harmful towards blood components due to the formation of soft protein corona around them. The reversible adsorption–desorption of proteins and the soft corona formation suggests that the MTX-LDH has flexibility and softness toward biological components, such as polymers, despite its inorganic nature.

## 4. Conclusions

A biological interaction between MTX-incorporated LDH (MTX-LDH) and blood components was carried to elucidate the blood compatibility of the MTX-LDH colloidal drug-delivery system. It was confirmed that the MTX-LDH had high RBC compatibility, showing insignificant hemolysis and a negligible effect on cell viability. The optical and scanning electron microscopies showed that the shape of RBCs did not change upon the administration of a very high concentration MTX-LDH. Furthermore, from the SEM and AFM studies, MTX-LDH was found to take advantage of RBC hitchhiking by attaching to the surface of RBCs. In order to comprehend the mild interaction between MTX-LDH particles and RBCs, the colloidal behavior of MTX-LDH in the plasma condition was examined. The zeta potential and dynamic light scattering study revealed that the surface of MTX-LDH could be readily covered by proteins and the colloidal size could be homogeneously maintained. Quantitative analyses of fluorescence quenching revealed that the strong interaction between MTX and proteins was reduced by the LDH shell, which could prolong the circulation time and enhance cellular uptake. Furthermore, the reversible adsorption and desorption of proteins on MTX-LDH were verified by the QCM assay. Through the reversible adsorption of proteins on MTX-LDH in a controllable manner, the MTX-LDH particles could acquire high compatibility with blood components. Therefore, it could be suggested that LDH-based delivery systems are highly recommended for intravenous administration with possibly long-term circulation.

## Figures and Tables

**Table 1:** Quantitative treatments of the plasma fluorescence quenching effects by MTX-LDH and LDH.

Quenchers	Type of Quenching	Regression Equation	Number	R^2^	K_SV_	K_S_
MTX-LDH	Stern–Volmer	N.A.	N.A.	N.A.	N.A.	N.A.
LDH	Stern–Volmer	*F*_0_/*F* = 1.01 + 0.12[*C*]	8	**0.9043**	0.116	N.A.
	Perrin	Log(*F*_0_/*F*) = 0.0064 + 0.049[*C*]	8	0.8591	N.A.	0.049
	Polynomial	*F*_0_/*F* = 1.00 + 0.20[*C*] − 0.054[*C*]^2^	8	**0.9785**	N.A.	N.A.

## Data Availability

The data presented in this study are available upon request from the corresponding author.

## Supplementary Materials

The following supporting information can be downloaded at: https://www.mdpi.com/article/10.3390/ma16196523/s1, Figure S1: In vitro (a) hemolysis after incubation with MTX-LDH and CO_3_-LDH suspension with 1, 5, and 10 mg/mL in human blood for 0.5 h and 24 h, and (b) the number of viable blood cells after incubation with MTX-LDH and CO_3_-LDH suspension with 1 mg/mL and 10 mg/mL in human blood for 0.5 h and 24 h. Figure S2: SEM images of LDH (a) and MTX-LDH (b) with the corresponding size distribution. Particle sizes were obtained by Nano Measurer 1.2 software in SEM images. Mean particle size and standard deviation were obtained by a *t*-test in Origin. Figure S3: SEM images of red blood cells’ incubation with MTX-LDH (10 mg/mL) after 3 h. Figure S4: (a) AFM images of MTX-LDH dispersed in water as a morphology map, and (b) cross-sectional height profile of the line in the image in (a). Figure S5: (a) Zeta potentials and (b) particle size distribution of LDH and MTX-LDH in neutral aqueous suspension. Figure S6: Fluorescence quenching of human plasma by (a) MTX-LDH and (b) LDH, fitted with the Stern–Volmer equation, Perrin equation, and polynomial equation.
