# Supplementary material for: Blood Compatibility of Drug–Inorganic Hybrid in Human Blood: Red Blood Cell Hitchhiking and Soft Protein Corona
Source: Materials (Basel). 2023 Sep 30;16(19):6523. doi: 10.3390/ma16196523 (PMC10573551; doi:10.3390/ma16196523)
Supplement: Supplementary file 1 [file materials-16-06523-s001.zip › materials-2578969-supplementary.pdf]

materials

Supplementary Materials

Blood Compatibility of Drug-Inorganic Hybrid in Human Blood: Red Blood Cell Hitch-Hiking and Soft Protein Corona

Jing Xie [1,§], Hyoung-Mi Kim [2,§], Kai Kamada [3,*] and Jae-Min Oh [1,*]

[1] Department of Energy and Materials Engineering, Dongguk University-Seoul, Seoul 04620, Republic of Korea; jingxie@dongguk.edu
[2] Biomedical Manufacturing Technology Center, Daegyeong Division, Korea Institute of Industrial Technology (KITECH), Yeongcheon-si 38822, Korea; annabb@kitech.re.kr
[3] Department of Materials Science and Engineering, Faculty of Engineering, Nagasaki University, 1-14 Bunkyo-machi, Nagasaki 852-8521, Japan.
* Correspondence: kkamada@nagasaki-u.ac.jp (K.K.); jaemin.oh@dongguk.edu (J.-M.O.)
§ Equally contributed.

1. **Hemolysis and cell count assay**
2. **Scanning electron microscopy**
3. **Atomic force microscopy**
4. **Dynamic scattering (DLS) and electrophoretic light scattering (ELS) measurements**
5. **Fluorescence quenching assay**

1. Hemolysis and cell count assay

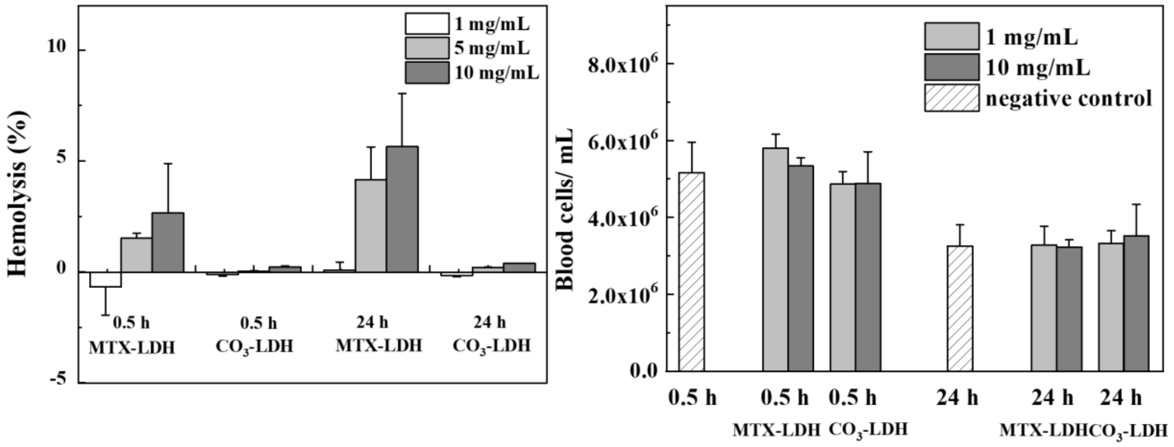

Figure S1. In vitro (a) hemolysis after incubation with MTX-LDH and CO3-LDH suspension with 1, 5, 10 mg/mL in human blood for 0.5 h and 24 h; (b) The number of viable blood cells after incubation with MTX-LDH and CO3-LDH suspension with 1 mg/mL, and 10 mg/mL in human blood for 0.5 h and 24 h.

2 SEM

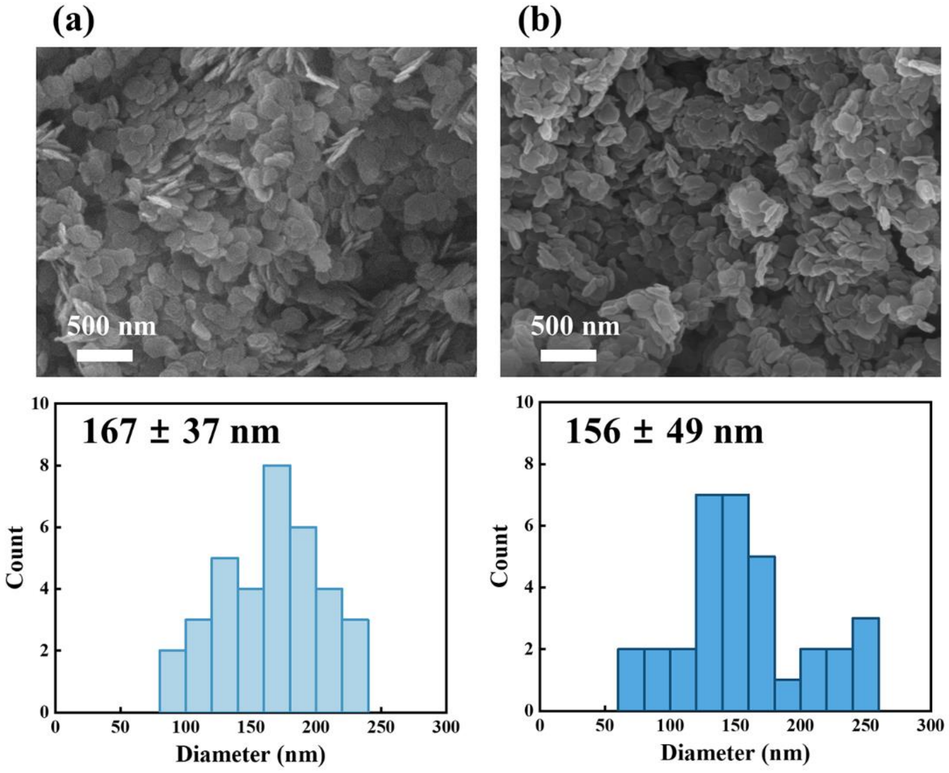

Figure S2. SEM images of LDH (a), and MTX-LDH (b) with the corresponding size distribution. Particle sizes collected by Nano Measurer 1.2 software in SEM images. Mean particle size, and standard deviation obtained by t-test in Origin.

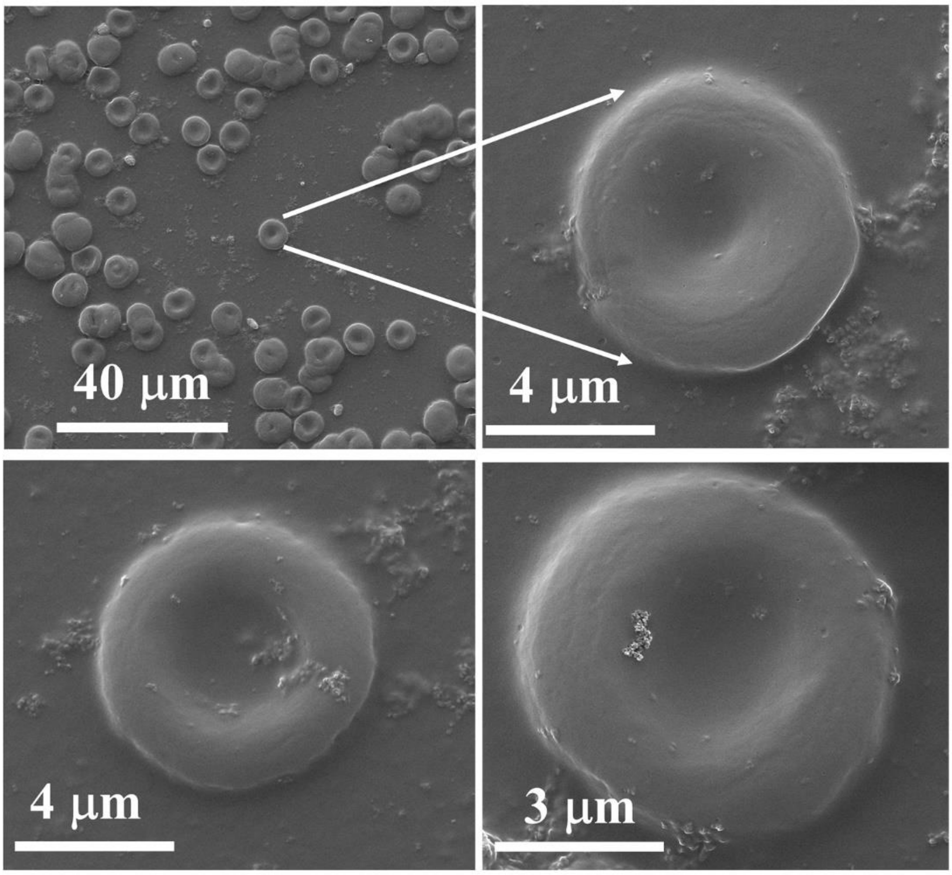

Figure S3. SEM images of red blood cells incubation with MTX-LDH (10 mg/mL) after 3 h.

3 AFM

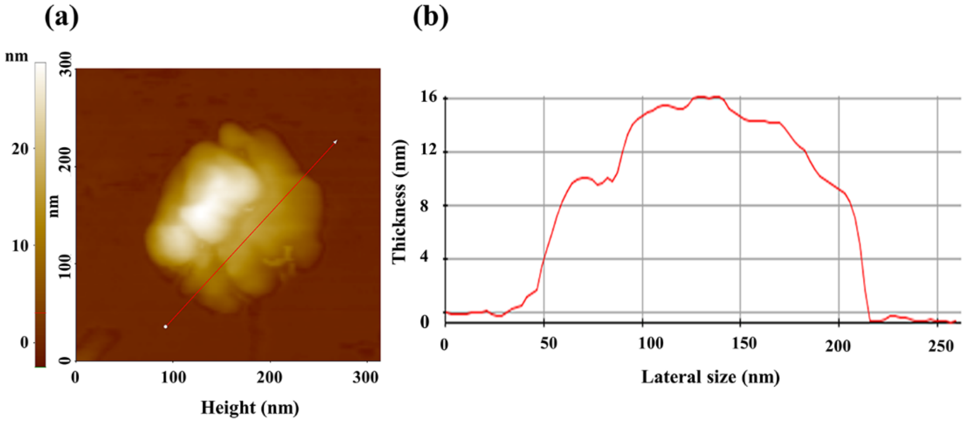

Figure S4. (a) AFM images of MTX-LDH dispersed in water as morphology map, and (b) cross-sectional height profile of the lined part of image.

4 Dynamic scattering (DLS) and electrophoretic light scattering (ELS) measurements

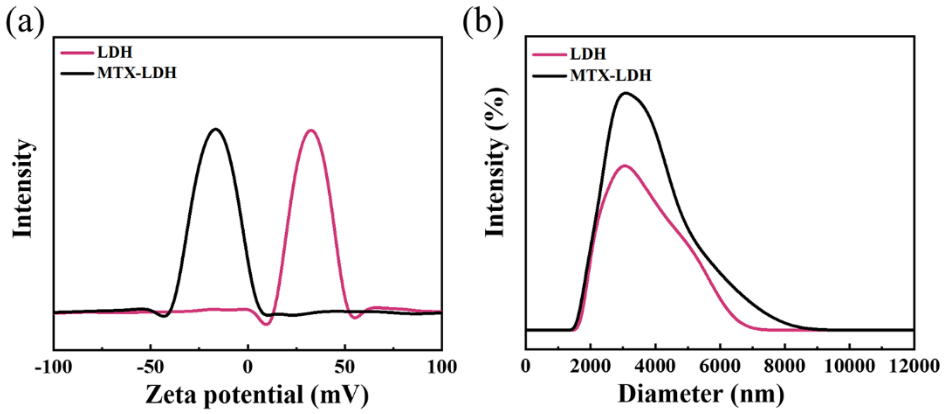

Figure S5. (a) Zeta potentials and (b) particle size distribution of LDH, and MTX-LDH in neutral aqueous suspension.

5 Fluorescence quenching assay

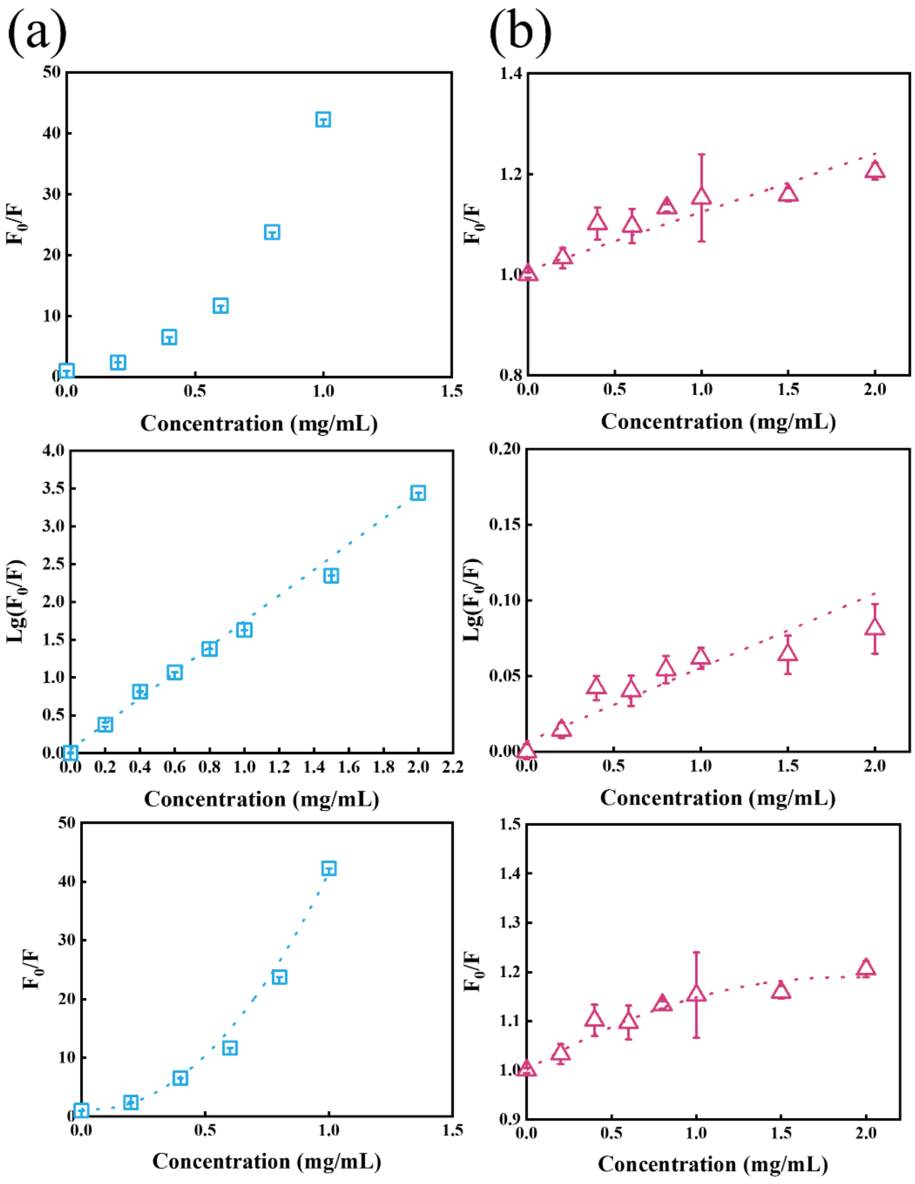

Figure S6. Fluorescence quenching of human plasma by (a) MTX-LDH, and (b) LDH, fitted with Stern-Volmer equation, Perrin equation, and polynomial equation.
